# Status Construction During COVID-19: Antibody Positive People's Rising Prestige

**DOI:** 10.3389/fsoc.2020.576827

**Published:** 2020-12-22

**Authors:** M. D. R. Evans, Jonathan Kelley, Sarah Kelley

**Affiliations:** ^1^Department of Sociology, University of Nevada, Reno, NV, United States; ^2^Interdisciplinary Social Psychology Program, University of Nevada, Reno, NV, United States; ^3^Nevada Agricultural Experiment Station, University of Nevada, Reno, NV, United States; ^4^International Survey Center, Potato Point, NSW, Australia; ^5^Department of Data Science and Innovation, Child Trends, Bethesda, MD, United States

**Keywords:** COVID-19, status construction theory, emerging inequality, status beliefs, immunity, antibody positive, status distinction, local context

## Abstract

The protracted COVID-19 crisis provides a new social niche in which new inequalities can emerge. We provide predictions about one such new inequality using the logic of Status Construction Theory (SCT). SCT, rooted in Expectations State Theory and from there developed by Ridgeway and colleagues, proposes general hypotheses about how new inequalities arise through process of interaction at the individual level: an unordered categorical difference becomes attached to a cultural value that gives one category more value than the other; social scripts concerning it emerge; small elements of assertion and deference creep into more and more encounters that an individual participates in, hears about through networks, and learns about via social and conventional media. The categorical difference begins to morph into a hierarchical status distinction. Through these mechanisms, individuals develop “status beliefs” that most people in their communities endorse the status distinction. Although they may or may not endorse the distinction personally, they believe that most people do so and they find that the path of least resistance socially is to enact the scripts that affirm the higher status/prestige of the favored group. We apply Status Construction Theory to the categorical difference between Antibody Positives (who have been tested for IgG antibodies) and Others (everybody else). Using the general logic of SCT and specifically developing applications of its key propositions, we predict that the categorical difference between Antibody Positives and Others will transition to a status distinction and propose testable, falsifiable hypotheses about each step of the process.

## Introduction

### COVID-19 and the Potential Emergence of New Inequalities

The COVID-19 pandemic of 2020 unleashed the Grim Reaper to slash his way across the globe and the infectious nature of the disease has pervasively disrupted the ordinary micro-encounters of which social life was built. Like flood, fire, hurricane or earthquake, the early stage of the crisis demanded an immediate solidaristic response, but, unlike them, the threat it poses endures a long time. This long duration provides fertile ground for new inequalities to emerge.

How would this happen? Consider the micro-processes of social interaction as a route whereby new hierarchies emerge. Specifically, Status Construction Theory posits that when a publicly knowable, nominal social difference—a characteristic or trait that people can recognize—gets associated with hierarchical placement on a cultural value, then, through social encounters in which people in one category assert dominance and people in the other category offer deference, that difference morphs into a hierarchical social distinction, a status distinction. Such encounters influence not only the beliefs of the direct participants, but also the beliefs of observers and hearsay recipients. Status construction theory explains how, given the right structural conditions, individual-level interactions and perceptions crystallize around an established cultural value to turn an inequality-neutral difference into a status distinction (Ridgeway et al., 2009; Ridgeway, 2018).

Nomenclature: In other literatures, this would be called a prestige or honor distinction (Treiman, 1977; Goode, 1978). “Status” in this context differs from ordinary language in that it is always strongly evaluative and refers to location on a hierarchy, not merely to nominal categorical differences (in contrast, e.g., to ordinary language's “marital status” etc.).

### Testing Positive for IgG Antibodies: An Emerging Status Distinction?

Applying Status Construction Theory to the COVID-19 crisis implies that, during the course of the epidemic, people who test positive for IgG antibodies (showing that they have been infected, recovered, and so are now immune at least for some time and therefore no danger to themselves or to others—“Antibody Positives” as we will call them), will emerge as a higher status group compared to Others who lack proof that they belong to the favored group, barring the several conditions discussed below that would derail the process. This has a strangely ironic flavor because people who “do all the right things” and socially distance rigorously are less likely to get COVID-19, therefore less likely to be have the IgG antibodies, and hence, according to the theory, will find themselves in the lower status group on this distinction!

Moreover, although some Antibody Positives have entered that category because of brave and altruistic dedication as health care providers, many others got COVID-19 because they flouted authoritative guidance or, in some cases, laws. This puts them in a morally ambiguous position, rather like blockade runners who are lauded for their courage and for what they supply, yet resented for the profiteering they do on the side. Common sense says that this ambiguity will prevent antibody status from emerging as a status distinction with the Antibody Positives gaining higher status than others. That makes the prediction of rising status to Antibody Positives a sharper test of Status Construction Theory.

## Why Status Construction Theory?

Status Construction Theory demands our attention here, because it concentrates specifically on the processes through which hierarchical status distinctions arise from knowable social differences not previously hierarchical. Many theories and vast amounts of research concern how inequalities and hierarchical status distinctions are maintained, and some focus on how brute power is converted into authority, but few focus on the emergence of a status distinction in the absence of a prior power or resource differential. The focus on emergence or maintenance of a status distinction matters because the processes of influence may be very different: e.g., the imperatives that stimulated the emergence of an aristocratic warrior class in medieval England are very different from those that support the continuance of the mild-mannered hereditary peerage of the twenty first Century.

The theories that do focus on change often neglect to theorize the specific mechanisms whereby new status distinctions emerge: Classic Marxist theory of change focuses on structural conditions and makes assumptions about within-class interactions concerning the emergence of a class for itself, while neglecting to problematize interactions between groups (Marx and Engels, 1848 [1972]). Classic Durkheimian theory of change focuses on interdependence of groups and individuals rather than hierarchy (Durkheim, 1961 [1893]). Homans and the social behaviorists posit that, without further mediation, people will accept hierarchy because that is rewarding, but not how the hierarchy emerges (Homans, 1974).

By contrast, Weber's work on status groups incorporating structure, interactions within and between groups with an emphasis on hierarchy/ inequality, and resources (Weber, 1946 [1958]) forms the foundation for Expectations States Theory of interaction processes in established, functioning social organization (Berger et al., 1972; Ridgeway et al., 1985; Walker et al., 1986; Zelditch, 2018). Status Construction Theory extends and revises Expectations States Theory to focus on the process whereby a neutral social difference morphs into a hierarchical social distinction through the emergence of status beliefs from encounters in local contexts (Ridgeway, 1991, 2018; Ridgeway et al., 1998, 2009; Ridgeway and Erickson, 2000; Correll et al., 2017).

The theory was developed in the context of a systematic program of experimental laboratory research, but it has strong implications for behavior “in the wild,” as we hope to show below. The idea of extending the scope of Expectations States Theory or Status Construction Theory beyond the lab to “real world” social situations is not novel to this paper, but has been cogently proposed in prior research (Jasso and Rossi, 1977; Heng et al., 2018), although these applications have not been numerous. What is new here is the opportunity to observe “in the wild” whether a new status distinction emerges from a categorical distinction in a situation in which whole societies are coping with a novel threat from which some are safe (for themselves and to others), but others are not: The coronavirus pandemic. More of a stretch is moving from competence as the cultural value previously studied in Status Construction Theory to another cultural value, health/ survival. This is justified by taking seriously SCT's more general claim that existing cultural values (not just competence) can form the axis of status differentiation.

### Basics of Status Construction Theory

Our discussion of Status Construction Theory just below closely follows the work of Ridgeway and colleagues (Ridgeway, 1991, 2018; Ridgeway et al., 1998, 2009; Ridgeway and Erickson, 2000; Correll et al., 2017). Status Construction Theory grew out of Expectations States Theory (Wagner and Berger, 1993; Berger and Webster, 2018) and so also draws on concepts and postulates originally developed there, sometimes with slightly different labels.

Status Construction Theory holds that humans have a strong tendency to build hierarchy and that hierarchies derive legitimacy from their real or imaginary connection to a cultural value. Competence is the cultural value mainly examined in prior theory and research on Status Construction. Here, we hope to show that a directly parallel argument can be made about how the shared value of health will generate a status distinction, specifically who will or will not infect others and the accident of who is (or is thought to be) a safe contact for others during the epidemic. Their location on the certified safe/non-infectious side of the “safe or not safe about Covid-19” dichotomy will raise the status of Antibody Positives (people who have proof of being tested positive for the IgG antibodies that demonstrate a past infection from COVID-19), despite the fact that they are likely to have been less agentic in striving to avoid the infection. By contrast, although people who rigorously practiced precautionary behaviors to avoid COVID-19 more closely conformed to the injunctive norms promulgated by legitimate authorities and widely accepted in the community, their “reward,” nonetheless is to fall into the group of “Others” —all who lack proof of IgG antibodies—and to reap lower status as the Antibody Positive status distinction emerges.

An important feature of Status Construction Theory is the role of “status beliefs.” A “status belief” is a widely shared perception that most people in their local context respect members of the favored group (we might call it the A-team), but have less respect for the lower ranked group (the “B-team”). This differs importantly from self-interested beliefs because both members of the “A-team” and members of the “B-team” accept that most people in their community have greater respect for members of the A-team. “Status beliefs” are key to the theory, because distinctions crystallize around them and people organize their behavior around them: They become customary and link into group stereotypes (Ridgeway, 2018). They enhance the predictability of role partners' behavior of assertion and deference in direct encounters and hence increase existential security. Note that one need not personally endorse a status distinction in order to acknowledge that most people do. “Status beliefs” are rather general vertical axes along which positions or characteristics can be arrayed hierarchically, including a high vs. low ranking of a dichotomous characteristic, and which tap into deep cultural values.

The emphasis here is on “third-order” beliefs—one's perception of what “most other people” think about an issue (Correll et al., 2017). In this paradigm, first-order beliefs are your personal beliefs—what you think is best, how you would ideally order groups or actions, the status you accord to different groups. Second-order beliefs are your perceptions of what specific others think is best, the status order they endorse on a particular dimension (Troyer and Younts, 1997; Webster and Whitmeyer, 1999). Both first-order and second-order encounters can contribute to third-order beliefs—one's perception of the majority opinion. in situations where legitimate authorities (Zelditch, 2018) publicly weigh in on the issue, their influence could operate through third-order beliefs, because one expects the majority of people to conform to the legitimate authorities' position (Johnson et al., 2006; Correll et al., 2017), or through first-order beliefs, through authoritative moral reasoning conforming your moral views to those of a legitimated authority (Tipton, 1982). Moreover, explicit and implicit messages from media can also influence third-order beliefs and expectations.

In terms of the Antibody Positive thesis, the claim is that as people observe and experience in person, hear about through social networks, and ingest from media encounters granting higher status to Antibody positives, acceptance of the Antibody Positive status distinction will become a status belief, with both Antibody Positives and Others perceiving that most people grant higher respect and esteem to Antibody Positives.

### The Core Value and Its Connection to Antibody Status

In this instance, the core and enduring cultural value is that life is better than death and hence that health is better than sickness carrying a risk of death. Stemming from this core value is a more specific “axis of difference,” the attitude toward COVID-19: Avoiding it is a “good” and catching it is a “bad.” Hence, people who are not infectious, Antibody Positives, will be higher in public estimation than people who might be infectious, Others. Observing encounters will show Antibody Positives eliciting positive and grateful, possibly somewhat awed, feelings: They are pure and clean (Douglas, 1966; Douglas and Wildavsky, 1983). This especially elevates the self-concept of people at the bottom of the A-team, but increases their status anxiety (Jasso, 2001). By contrast, people who might be infectious (the “B team”) are potentially dangerous, so they all come to be treated as though they were dangerous. The safe feeling that Others experience in the presence of Antibody Positives also signals Others' vulnerability and the (high status) perceived invulnerability of the Antibody Positives. Increasingly, Antibody Positives will express dominance in encounters with Others, and the Others will increasingly defer to them. A counter-hypothesis would be that Antibody Positive people will be seen as morally contaminated or damaged by their prior contact with COVID-19, so they will be treated with revulsion/ repulsion by others and will have less influence on public policy than other people do.

What knowable characteristic differentiates the safe A-team members from the potentially contagious B-team members? In more specific terms, the concrete “axis of difference” of interest is the dichotomous characteristic of whether people could transmit COVID-19 to the people they interact with. As adumbrated above, those who have been tested for the IgG antibodies, and hence certified by legitimated authorities as having them, are high status on this dimension and others are low. Although medical authorities are still explicitly cautious about the degree of safety indicated by the IgG antibodies, it seems likely that the general public will see COVID-19 as analogous to other flu and flu-like infections where immunity is conferred at least for the season and possibly longer (depends on the speed of mutation).

Antibody Positives do not look different from Others, so, at the moment, there is no visually distinguishing feature, no immediately apparent “status marker” (in the language of Status Construction Theory) or status cue in the language of Expectations States Theory (Wagner and Berger, 1993; Berger and Webster, 2018). Hence, one issue is how is antibody status demonstrated? Demonstration of status via status cues is tied up with legitimacy. National governments have been debating the use of “immunity passports”: government-endorsed medical certifications that the person has tested positive for the IgG anti-bodies, is an “Antibody Positive” (Phelan, 2020). Moreover, the ethical acceptability of using IgG antibody status to enhance one's resume or boost the appeal of one's business is being debated in social media. For example, public discussions have argued the pros and cons of people beginning to use IgG antibody status as a qualification, e.g., as a host for overnight stays, as an attraction to their store or salon, etc. Because of the lack of visual distinctiveness, the only reliable indication is certification by the (government backed) medical system that one is Antibody Positive. Antibody Positives are likely to provide indicative status cues (explicit signals of their status) by proclaiming their status, or more subtly to provide expressive cues (signals that point to their status but do not explicitly proclaim it) that draw attention to their status, e.g., by mentioning in conversation how great the medical personnel were during their COVID-related hospital stay (Wagner and Berger, 1993).

## More on the Two Groups: Antibody Positives and Others

### Antibody Positive People (Who Have Proof That They Have Been Tested)

Antibody Positive people can go anywhere and associate with anyone without risk to themselves. They are immune to re-infection and so could go about their lives without fear of catching COVID-19 (we do not now know how long, but that will be known before long). That tantalizes them with the prospect of freedom to proceed with normal social life and to pursue new opportunities without violating the strong social norm against self-harm. That freedom is precious because people deeply value interaction with others and suffer from social deprivation (some more than others). Requiring them to take precautions that curtail their social life and economic opportunities in order to create or maintain social solidarity with the potentially risky Others is requiring a major sacrifice.

Moreover, because Antibody Positive people do not pose an infection risk to anybody else, they will begin to develop a sense of superiority about their employability and entitlement to lead normal lives, thus developing first-order beliefs and expectations about their higher status. This, in turn, because of the centrality of the health value and because others begin to defer to them, will begin to take on moral overtones: Not just higher on the totem pole, but worthy of the place. Outsiders to the group are less worthy. This will be aggravated by the fact that most Antibody Positive people (except the asymptomatic ones) have passed through the ordeal of a terrifying and life-threatening illness to receive their privileged status. Ordeals associated with status transitions are common in human societies (e.g., the excruciating “Sun Dance”), so it seems likely that the experience of the ordeal will reinforce Antibody Positive people's sense of being special.

It is an easy segue from being special to being deserving. Because entry into the group (to date) has been involuntary with a substantial random element, and humans are desperate to make meanings, members of the group are likely to develop a sense of being “chosen,” that they have some special hidden virtue that brought them into the group. The feeling of entitlement will lead to them being assertive in interactions with Others and they may feel that their direct experience with the disease gives them a special authority which entitles them to extra influence in policy making concerning the epidemic. They will also have self-interested reasons for making claims to privilege, but, according to Status Construction Theory, if they perceive that those claims to their group's entitlement to respect and influence are widely accepted in the broader community (third-order beliefs and expectations), that perception will intensify their assertive, dominant behavior above and beyond the self-interest influence *per se*.

Because membership is long term—we do not yet know if it is for a season like an ordinary flu or lifetime immunity like measles—that provides a further basis for making status claims. Relationships build over time, so an enduring position is more likely to form the basis for feelings of commonality and the potential to act on them. In the language of Expectations States Theory, repeated encounters and multiple encounters with the same outcome strongly reinforce first, second, and third order beliefs and expectations (Wagner and Berger, 1993; Webster and Whitmeyer, 1999). If Antibody Positive people begin to form networks (perhaps on the internet or perhaps in person), they are likely to inflame each other's resentment of blanket restrictions: There is no medical/health reason they cannot go where they want with whomever they want. They will begin to push for selective restrictions, because that will reinforce their “pure” higher status, enhance their employment opportunities, and allow them to enjoy the pleasures of everyday social intercourse. They will strongly support social closure restricting public contact jobs and, possibly on-site work more generally, to Antibody Positives. Shops and stores may soon be advertising that all their employees are Antibody Positive. According to Status Construction theory, all these feelings and claims will be intensified by perceiving that “most people” accept these claims, a status belief, also called a third-order belief.

It is important to note that Status Construction Theory is a “net” or *ceteris paribus* theory” that does not dispute that self-interest and other social forces may also influence whether people personally endorse the status difference (first order effect), but, instead, makes the claim that, above and beyond other influences, the emergence of status beliefs (on the part of both the A-team and the B-team) perceiving that most people accept the status distinction will influence individuals' future behavior. Thus, the claim is that the third-order beliefs and expectations will have a substantial effect on action: Antibody Positives who perceive that the majority of people grant their higher status position will become more assertive and Others who perceive that the majority of people accord them lower status will become more deferential.

### Others (People Who Do Not Have Proof of Antibodies)

“Others” form a heterogeneous group: Some have diligently practiced precautionary measures such as social distancing and hand washing and so avoided catching COVID-19; some have missed it by luck; some have it now; some have had it but not been tested; some will have had it and not even know they have had it. Regardless of why they are Others, in encounters with an Antibody Positive, they will be expected to defer and show respect to the Antibody Positives. They will also hear about other such encounters through their social networks and media, so Status Construction Theory posits that they will develop status beliefs endorsing the antibody status distinction, the higher status of Antibody Positives and lower status of Others. In contexts where encounters between Antibody Positives and Others are rare, Others will be less likely to perceive a status distinction favoring Antibody Positives. The self-concepts of diligently precautionary, righteous Others will be higher where Antibody Positives are rare, because they will experience the moral glow of doing the right thing and rarely, if ever, experience, see, or hear about status loss associated with their good behavior.

The group most likely to experience initial resentment about the emergence of a status distinction favoring the Antibody Positives will be those diligently practicing precautionary measures, the “Righteous Others.” Most of these practices are personally costly to most people, but have brought the subjective reward of “doing the right thing.” By contrast, those who have had Covid-19 are at least slightly tainted with the possibility that they indulged in pleasures the righteous have forgone and thus failed in their responsibility to protect the community by practicing precautionary measures. Indeed, early on, it is possible that the Antibody Positives will have lower status because of the moral contamination of misbehavior and contagion. As the status distinction emerges, for righteous Others to see the Antibody Positives socially rewarded with higher status is likely to be galling. A key prediction of Status Construction Theory is that even these diligent practitioners of precautionary behavior will experience and hear about micro-encounters in which the Antibody Positives are accorded more respect and influence and that this will, in time, build their status belief, their third order inference that most people endorse the status distinction granting Antibody Positives higher status.

Because the key status distinction will be shifting from differentiating the people who take socially approved precautions which have been legitimated by recognized authorities from their slothful or self-indulgent peers, to differentiating the Antibody Positives from others, the status difference between righteous and unrighteous Others will fade, most likely with the status of the righteous falling to that of the unrighteous (on the tendency in the presence of status differentiated groups to treat all groups members as though they were the modal or median member see Jasso, 2001). Alternatively, a three-status group system could emerge, with the Antibody Positives on top, the righteous Others in the middle, and the unrighteous Others at the bottom. But it seems more likely that the distinction between the two groups of Others will fade because behavior is fluid enough that the unrighteous could easily “pass” for particular events or opportunities and there is, at least at present, no publicly visible or authoritatively certified halo, no visual status marker (indicative status cue), that attaches only to those who have diligently practiced precautions for a long time.

The Others group also includes people who do not diligently practice precautionary measures and so they may be at risk themselves and one cannot be sure that they are safe for others. This includes people living very low-risk lifestyles; people living in very low-risk contexts; people at higher risk, but simply spared by chance, the lucky ones who do not diligently practice precautionary measures but, by chance, have not caught COVID-19; and people who have had COVID-19 but not been tested to certify their status as Antibody Positive. They will all be treated as outside the favored group in encounters with Antibody Positives or with gatekeepers selecting for Antibody Positives, and hence should all develop the status belief (third order belief and associated expectations) that most people accord higher status to Antibody Positives. But there are also likely some differences within this group. People with living in low risk contexts are likely to have few encounters with Antibody Positive people (because these contexts will mostly be places where COVID-19 has been rare), so they will be less likely to experience, see, or hear about encounters in which the Antibody Positive person is treated as higher status. In other words, they will have fewer first-order and second-order experiences in which Antibody Positives are accorded higher status That in turn will mean that they will be less inclined than their peers in higher-risk contexts to accept the status belief (the third-order belief) that most people think that Antibody Positives are higher status, and hence the third-order expectation that most people will accord Antibody Positives higher status. People who are just lucky will have an average number of experienced, seen, or reported encounters in which Antibody Positive people are treated with deference, so they will develop an average level of status belief (third-order belief) that most people accord Antibody Positives higher status.

Some of the members of the Others group will have recently tested negative for currently having COVID-19 (and some of them are routinely tested). They will be certified by legitimated authorities as being, temporarily, safe for others and safe themselves. Hence, they have an indicative, albeit not normally visible, status cue/ status marker of non-dangerousness to others. The test certification will become socially relevant when they are observed using their test certification to enter workplaces or other restricted areas. Membership in this category is, however, precarious because it is temporary. COVID-19 infection windows may be as short as 4 days, so even if the “short windows” are less common than the longer windows, the uncertainty means that all in this category will be treated for the purposes of privilege as though the short window were correct (e.g., access to work). For example, a hospital might entitle someone to work on site for 3 or 4 days after their test before requiring a re-test (assuming accurate, near-instantaneous results). These people are likely to be located in contexts where COVID-19 has a high incidence, so they will experience, witness, and hear about many encounters between Antibody Positives and others and hence will develop status beliefs (third-order beliefs/expectations) that most people accord higher status to Antibody Positives. Because they are likely in a setting where COVID-19 is highly salient, the effect of the number of encounters may be amplified.

### In Transition

People who currently have COVID-19 will, provided they survive and are subsequently tested for antibodies, belong to this group. They will not yet have experienced encounters placing them at higher status, at the moment being treated as dangerous, yet in extreme danger, possibly with a whiff of moral contamination (Douglas, 1966) and they were previously Others. They may have previously had the experiences of dominance and deference that are the building blocks of status beliefs (third-order beliefs and expectations) as an Other and their current situation will not yet have given them the “top dog” experience, so their self-interest perceptions are likely to be unsettled and their status beliefs like those of not-currently-sick Others. They will perceive that most people perceive Antibody Positives as higher status (third order belief), but will not necessarily hold that status belief themselves (first order belief), although, as with the not-currently-infected Others, subjective pressures to resolve cognitive dissonances will likely lead to aligning their first order beliefs with their third-order beliefs. Moreover, the status of Antibody Positives is likely to be of low salience to them, unless hospitals evolve visually distinctive costuming for Antibody Positive personnel (indicative status cues) which would enhance the Antibody Positive status distinction in the eyes of the patients. Having Covid-19 is, of course, a transitory status, albeit of currently unknown duration.

## Application of Propositions from Status Construction Theory

### Foundations: Terminology

Before launching into the propositions, it is useful to review definitions of some key terms in status construction theory. A “**categorical difference**” (or categorical distinction) is a nominal difference without a difference in prestige/ status between the categories (i.e., the categories are nominal, not ordered, with respect to status). Here, the Antibody Positives who have proof of having tested positive for IgG Antibodies and Others (everybody else) are the two categories. The categorical difference is whether or not they have proof of having IgG antibodies. A “**status distinction**” refers to social categories (usually 2) that are ordered with respect to status. For brevity, the higher status category is sometimes called the “A Team” and the lower status category is called the “B Team.” Status Construction Theory posits how and under what conditions a categorical difference evolves into a status distinction. Our focus here is on the degree to which (and in what contexts) the categorical difference between Antibody Positives and Others evolves into a status distinction with Antibody Positives as the higher status category. A “**socially valid correspondence**” is established when an individual perceives that (1) most people in their local context accept a status distinction and endorse behavioral scripts that enact the status difference (third order inference and expectations), or (2) when a legitimated authority pronounces in favor of the distinction or acts more favorably to members of the A Team (see also Zelditch, 2018). In our example of the Antibody Positives, perceiving that “most people” in your community respect Antibody Positives and perceiving that Antibody Positives have disproportionate influence would establish or reflect a socially valid correspondence such that Antibody Positives have higher status. “**Goal objects**” are what people want. In our COVID-19 example, these include access to jobs, public venues, places of entertainment and leisure, and private gatherings. Influence on public events is also a goal object. “**Status typifications**” are the cultural scripts which people use as they enact status and hierarchy. For our purposes, they include both interpersonal scripting of assertive and deferential behaviors in direct encounters and scripting at one remove that rejects Others but preserves face for them by averting a personal interaction in which they would be rejected (for example, signage or language in job advertisements that specifies Antibody Positives only excludes Others, but also spares them rejection in a personal encounter). Here a “**Local Context**” is a setting in which interactions/ encounters occur. These can be neighborhoods, workplaces, and shops and places selling services, including online spaces, where at least some of the parties encounter more than once. Local contexts can vary in their social composition from those in which all persons present belong to the A Team, through settings including some members of each team, through settings in which all person present belong to the B Team. A “**Status Marker**” is a signal of esteem. It could be an act of deference or assertion; it could be something visibly demonstrable like a garment or luxury object; it could be something only the A Team members are allowed to possess. As of this writing, there are no visible status markers demonstrating the higher status of Antibody Positives, but they do possess universally accepted medical certification of their membership. Thus, the legitimacy of the medical establishment gives their certification legitimate force. In the language of Expectations State theory, medical certification of Antibody Positive status is an indicative statue cue (Wagner and Berger, 1993) that Antibody Positives are predicted to carry with them to display to gatekeepers (and to be seen by others displaying to gatekeepers) to gain entry to places that are closed to Others.

### The Propositions

Status construction theory has several propositions (Ridgeway, 2018).

1. Proposition 1. The categorical difference begins to evolve into a status distinction. The original:

*Local contexts that create a socially valid correspondence between a salient categorical distinction and status markers create a likelihood that their participants will form status beliefs about the categorical distinction*.

This application: (a) The establishment of a socially valid correspondence: Participants will observe that Antibody Positives (the categorical distinction with the status marker being possession of certification of IgG antibodies by medical authorities) are seen by most other people in their community as safe with respect to COVID transmission (the relevant cultural value being health and life) and hence are accorded specific privileges and influence. This correspondence may be established through first and second-order expectations in direct experiences in which one's expectations for oneself and one's perceptions of one's interaction partner's expectation for one come into play (Troyer and Younts, 1997; Webster and Whitmeyer, 1999). Moreover, reports from people in your social networks about such experiences. In addition, experiences or reports through media and encounters with governmental regulations and forms will inform your understanding of what “most people” think, thus establishing third-order expectations (Correll et al., 2017). Examples include, observing (or hearing friends report) Antibody Positive status as a condition for rental properties; observing (or hearing friends report) “employee wanted” signs specifying Antibody Positive status as a qualification for public contact jobs; being asked (or hearing that friends have been asked) to demonstrate Antibody Positive status as a condition of entry to public gathering places or events; being required to report your antibody status on forms; observing or hearing that Antibody Positive people draw attention to their status; observing or hearing that Antibody Positive people wield greater influence with city council members or other local politicians. These experiences will be strengthened at one remove by pronouncements and policies by city and state government (especially if they are trusted) favoring Antibody Positive people and by local media reporting instances from the above list. Typical experiences favoring or emphasizing the value of Antibody Positives in a local context may also filter through social network and media reporting to tilt respondents toward the perception that most people accept a higher status for Antibody Positive people than for others. The establishment will be strengthened if legitimated authorities such as the CDC and the FDA announce that Antibody Positive people are not contagious and that this is a condition that will hold for at least several months. (b) These experiences will shift respondents' perceptions in the direction of thinking that most people accept that Antibody Positive people are safe: that they are healthy and do not endanger others'health. As the process evolves, the cumulation of such experiences will lead individuals to adopt the status belief that most people in their local context accord higher status to Antibody Positives. We can extend the theory to suggest that living in a community where behavioral scripts enacting the status distinction are widespread is likely subtly to influence an individual to adopt the status belief that most people accept the status distinction favoring Antibody Positives, above and beyond the encounters the individual experiences, hears about, or attends to sufficiently to report.

2. The status distinction is further reified or collapses back into a nominal difference. The original:

*Subsequent local contexts in which the categorical distinction is salient (typically, encounters with others who differ on the distinction) and that confirm the correspondence between the distinction and status markers increase the social validity of the correspondence for the participant, while inconsistent, disconfirming experiences will undermine its social validity*.

This application: (a) Take the case that that no effective vaccine is yet universally available and that there are no announcements from legitimate authorities that undermine the correspondence of Antibody Positive status with health and safety. Friends and acquaintances who have become Antibody Positive mention receiving invitations that Others have not. There will be recurrence and increasing frequency and strength of incidents indicating the connection of Antibody Positive status with health and affirming the higher status of Antibody Positive people. These will strengthen the perception that most people accord more respect and esteem to Antibody Positive people. Lack of public outcry against Antibody Positive people agitating for greater privileges will reinforce the status belief. (b) Take the case that an effective vaccine is universally available; or that there have been announcements from legitimate authorities that Antibody Positive status does not confer immunity or otherwise undermining the correspondence between Antibody Positive status and health; or that there is a spectacular burst of infection that is (correctly or incorrectly) attributed to an Antibody Positive person; or that effective antiviral drugs are developed for COVID-19, so that catching it becomes a minor inconvenience rather than a mortal danger. In this case, employers and landlords may begin to resile from their discrimination in favor of Antibody Positive people and this will become widely known. Privileges limiting public events and venues to Antibody Positive people will be withdrawn (those events and venues may then be closed to all). Challengers may contest influence claims or assumptions of Antibody Positive people. Awareness of all these encounters will undermine the perception that most people accept a higher status for Antibody Positive people. Alternatively, another possible path obstructing the emergence of a status distinction favoring Antibody positives or dissolving one that has emerged would a governmental commitment to universal testing, such the universal weekly spit test proposed by Prof. Julian Peto and colleagues at the London School of Hygiene and Tropical Medicine (Walsh and BBC, 2020, July 17) which would show who among the Others is (temporarily) safe—those carrying their medical certifications of antibody positive status would no longer be the only ones with a “safe” status marker/status cue.

3. The greater the social validity, the stronger the perception of widespread endorsement of the status distinction.

The original:

*The greater the apparent social validity for a participant of a correspondence between a categorical distinction and status markers, the more likely participants will form strongly differentiated correspondent status beliefs about the distinction*.

The application: Strong, repeated announcements from legitimated authorities, affirming that Antibody Positive status demonstrates immunity and hence protects the health of anyone coming into contact with the Antibody Positive person will further strengthen the perception that this is a widely held view. This will also be true for local authorities such as state governors and city councils, provided that they are trusted. Similarly, as discrimination in favor of Antibody Positive people in public contact jobs, as tenants, and for likely-contact public events and venues becomes increasingly common and uncontested, it will come to be taken for granted and will be perceived as something that most people endorse (third-order belief). The longer discrimination has been in place, the more will people perceive it as having widespread acceptance. Should distinctive visual markers—indicative status cues—for Antibody Positive people emerge, all these effects will be amplified. As Antibody Positive people increasingly take their status distinction for granted, they will cease to apologize for it and will be increasingly confident about asserting it. As Others are seen to defer to these status claims, the perception that the status difference is widely endorsed will be strengthened (third-order belief and expectations flowing from that).

4. Spillover: Status beliefs formed in one context will spill over into other contexts.

The original:

*Actors transfer status beliefs formed in one context to future encounters with others who differ on the categorical distinction*.

The application: Perceptions of social consensus about the higher status of Antibody Positive people in different domains will be highly correlated (third-order beliefs). To the extent that these can be temporally located, an increase in an individual's perceived social consensus supporting employment discrimination in favor of Antibody Positive people in time 1, for example, will ramify by time 2 into that individual perceiving social consensus endorsing the higher status of Antibody Positive people in other domains such as housing, access to crowded events, and influence in public meetings. These will be effects net of actual interactions in those domains.

5. Role modeling deference.

The original:

*In a context in which the participants differ on the categorical distinction, an actor can spread status beliefs to other participants by treating another according to the status belief*.

The application: Any observed interaction in which an Other person defers to an Antibody Positive person or expresses esteem for them (verbally or non-verbally) increases the degree to which the observers perceive that the nominal difference between Antibody Positives and Others is a widely endorsed status distinction (third-order belief). These may be observed in person, reported through networks, or reported through conventional and internet media—many of the building blocks are first -order and second order beliefs. An Antibody Positive person who receives an instance of deference (e.g., getting a restricted job, getting a restricted tenancy, attending restricted events or venues) will act as though they are privileged and as though that privilege has wide public support: Bit by bit, they will assume dominance in interpersonal decisions, will assert themselves in public meetings, will endorse the wearing of distinctive garments or insignia to signal their status (status cues) while feeling confident that both Antibody Positives and Others support these status distinctions (third-order beliefs). Others may feel reluctant to openly disagree with Antibody Positives.

6. Structural conditions that favor the distinction.

*Given a correlation between the distribution of an influence biasing factor and a categorical distinction in a society, interactional processes will be sufficient to create widely shared status beliefs about that distinction that favor the categorical group advantaged by the factor*.

Several structural conditions, also called influence-biasing factors, will be important to the spread of a status distinction favoring Antibody Positives over Others. (a) Certification of Antibody Positive status by health authorities will act as a status marker/ status cue that will establish and solidify both the individual perception that Antibody Positives are healthy and safe interaction partners for all (first-order belief) and also the perception that this view is a widely shared consensus (third-order belief). By implication, that will brand all Others as unhealthy and unsafe, although, in fact, especially early in the process, quite a few of them may actually have had COVID-19 and have acquired the relevant antibodies, but have not been tested for them. (b) The existence of a market system for shopping will influence some owners to attract customers, and possibly raise prices, by advertising that all their public-contact employees are Antibody Positive. This will help build and reinforce the status distinction (third-order belief). (c) The same will hold for rental of housing in multi-occupant units ranging from duplexes to apartment buildings. (d) The same will hold for employment. Others are not a protected category under EEO legislation, so employment biases in favor of Antibody Positives will occur. People will hear about instances favoring Antibody Positives, or possibly experience them directly if they apply for a job that requires evidence of Antibody Positive status. First and second order beliefs will thereby be affected. For any job that requires working on site with others (whether public contact or out of public view), employers will have an incentive to look to certification of Antibody Positive status in making new hires to reduce their risks of being the source of a public outbreak or of having an outbreak among their workers and being shut down. (This does not apply to remote workers.) The employers may have to pay more for Antibody Positives. Some employers may hope to increase profits by not checking Antibody Positive status and paying their on-site workers less than employers who check. An implication is that Antibody Positive status will increase likelihood of being hired and increase pay only for jobs that require on-site interaction with the public or with others employees. No such changes will occur for people who work entirely remotely, so the status distinction may be more prominent among people working in blue collar and personal service jobs. All of these structural conditions will contribute to the emergence and solidification of third-order beliefs supporting a status distinction favoring Antibody Positives (Proposition 7 concerns processes in the absence of structural conditions favorable to the emergence of a status distinction, so it is not relevant here).

The potential segregation of leisure may sound farfetched, but there are already instances of it occurring with the sanction of governmental authorities, for example, Brazil restricting access to the spectacular vacation destination island Fernando de Noronha to Antibody positives (https://www.theguardian.com/world/2020/aug/30/brazil-island-fernando-de-noronha-reopens-tourists-covid-19). Displaying souvenir paraphernalia associated with such vacation destinations may become an expressive cue for Antibody Positive status.

### Awareness of Encounters and Immersion in a Local Context

A scope condition of Status Construction Theory is that members of the two categories encounter each other and interact, thereby enacting and observing respect and deference behavior. We will expect those direct, first-order and second-order interactions to be influential in the formation of status beliefs. But we will also allow for the possibilities of other routes of information about such encounters to influence third-order inferences about what the majority of people think. (A) Reports of such encounters from close network members, mainly family and friends. These very likely signal the status belief that the informant holds; if so they are a form of second-order encounter. (B) Environmental observation of signs and symbols indicating that privileges such as entry or employment will be restricted to Antibody Positives at particular establishments or events sounds like it might require separate interpretation, but such signs and symbols would not appear unless the persons or groups posting them expect compliance (an expectation of dominance). That, in turn suggests that the persons responsible for posting the signs and symbols assume that most others share their status belief. Hence, this can be seen as a form of third-order encounter (or possibly a second-order encounter if the entity presenting the sign or symbol is personally known to the observer). (C) Conventional and social media incidentally showing or reporting on dominance-and-deference encounters between Antibody Positives and Others in a manner consistent with the status difference will seem to assume that it can be taken for granted. This will contribute to the third-order belief that most people accept the status difference. (D) Moreover, we will also expect the local context to influence individuals adoption of status beliefs subtly: Above and beyond the information of which the individual is explicitly aware, the degree of acceptance of the status distinction in the individual's local context, the social climate, will influence the degree to which the individual adopts the status belief (Jasso, 2015) favoring the Antibody Positives.

## Summary of the Application of Status Construction Theory to the Emergence of a Status Distinction During the COVID-19 Epidemic

**Figure 1:**
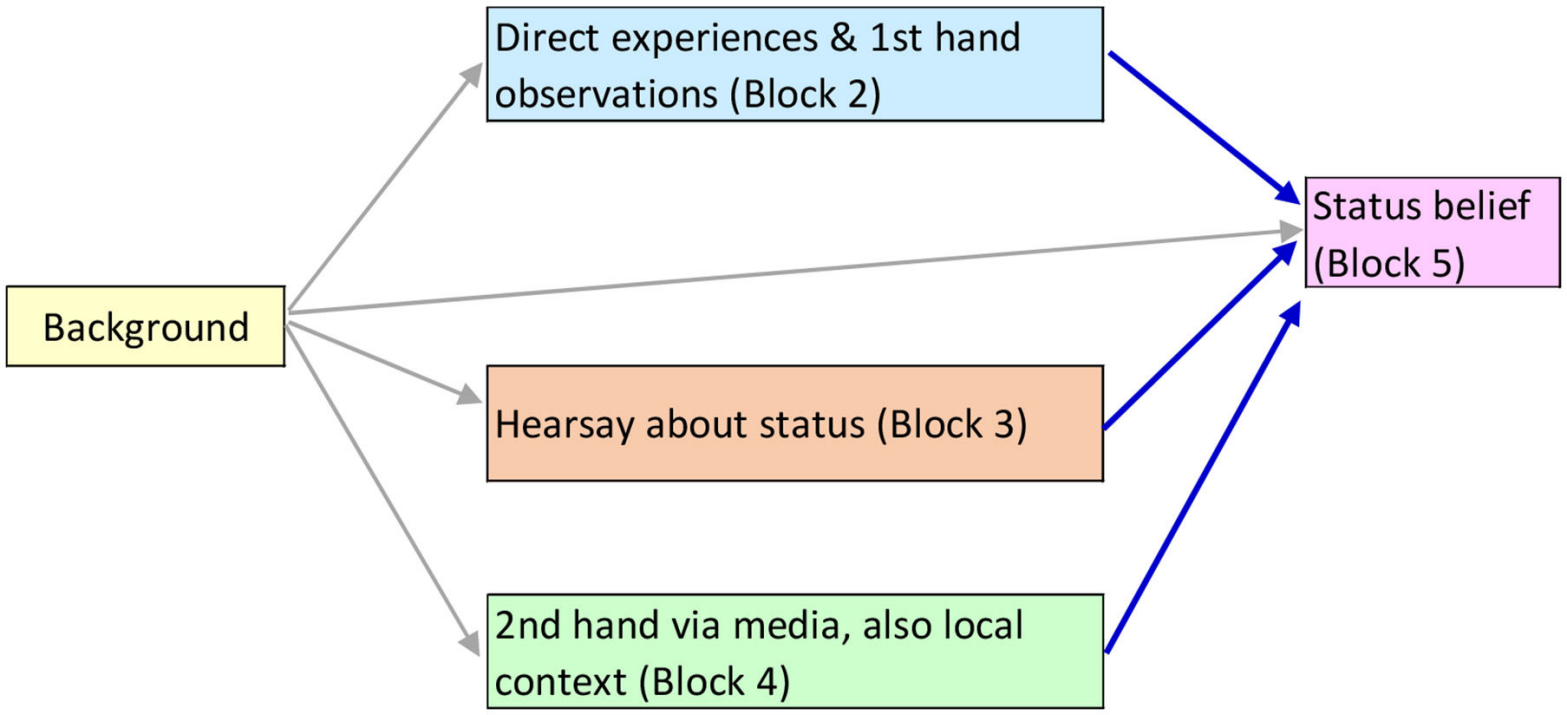
Schematic conceptual model of the emergence of a status belief that Antibody Positives are higher status.

We can summarize these arguments schematically in a conceptual model, as shown below (Figure 1). Note that the conceptual model adds in background variables, which for the purposes of the theory, are simply control variables and so are not discussed here. The theory was developed in experimental settings so control variables were not pertinent, but it is reasonable to extend the theory to a naturalistic “wild” setting by positing that the theory's propositions are net of other influences, in short that all the theory's claims are *ceteris paribus*.

**Figure 2:**
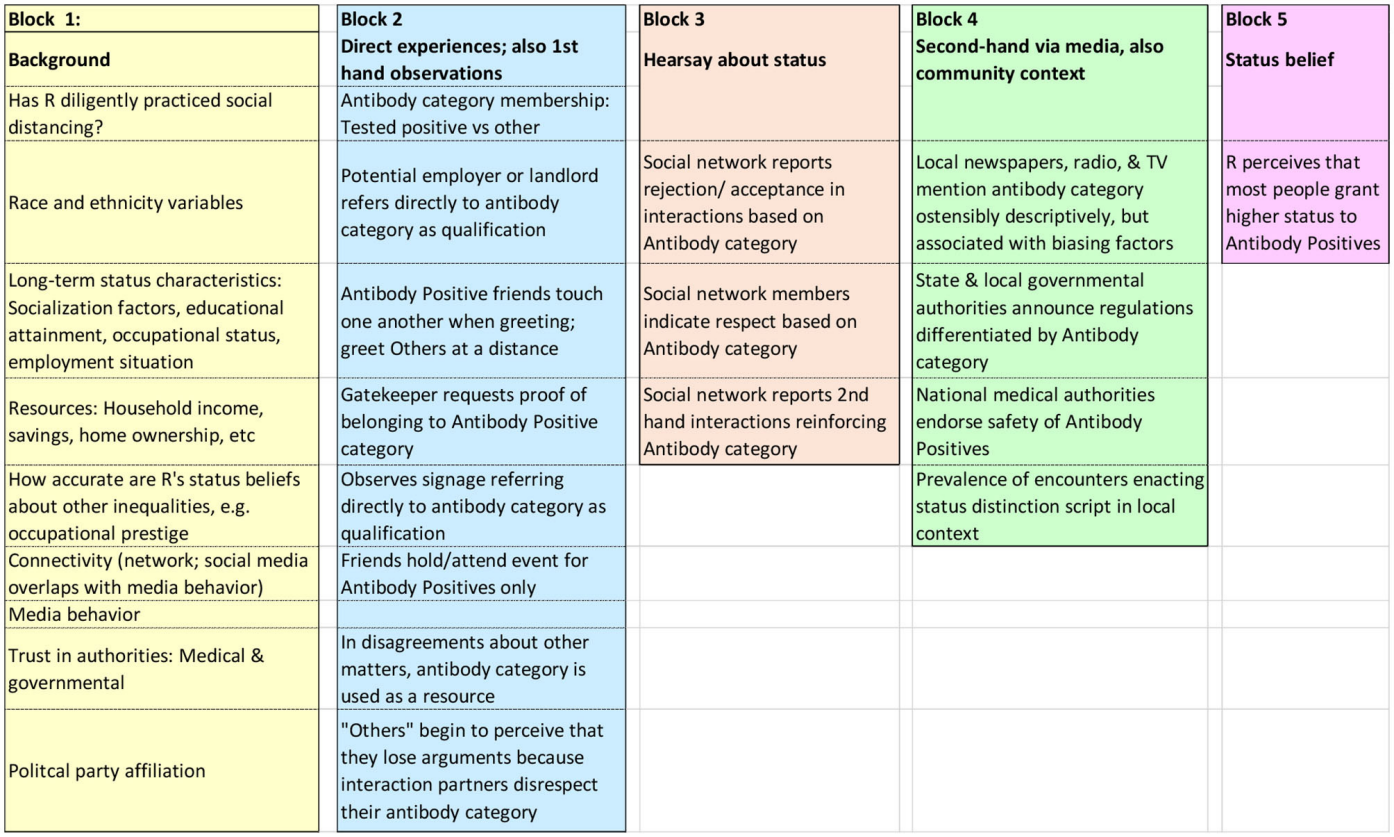
Listing of some candidate indicators of the influences shown in Figure 1.

To put some flesh on the bones of the schematic conceptual model, below are listed some of the indicators one might use to operationalize the concepts (Figure 2). Note that the blocks correspond to the conceptual model above. The causal order is shown in Figure 1. Figure 2 gives a bit more content to the blocks.

Note that the theory does not claim that Antibody Status negates the status inequalities included among the background variables (e.g., education, income, ethnicity, race, gender, etc.). Instead, the theory posits that Antibody Positive Status is emerging as an *additional* status dimension. This raises several new questions on which the theory is silent to date, namely how large is the gain in status for Antibody Positives and whether that gain is uniform across social groups, or whether different dimensions of status amplify or deflate each other. We take up these issues in the Discussion.

## Discussion

Thus, this paper proposes that Status Construction Theory can be applied to the categorical difference between Antibody Positives (people who have proof that they have tested positive for IgG antibodies) and Others (everybody else). Following the logic of Status Construction Theory leads to clear and strong predictions that, barring a set of dissolving events, (1) the categorical difference between Antibody Positive people and Others will transition to a hierarchical status distinction favoring Antibody Positives and (2) deference/dominance interactions will be experienced and observed that will lead individuals to develop status beliefs that most people in their local contexts accept the status distinction. A set of operationalized hypotheses concerning this transition has been registered with the Open Science Framework (DOI suppressed pending review.) Note that these predictions are for net differences, they do not presuppose that other pre-existing status distinctions will evaporate. Instead, the theory proposes that Antibody Positive status will emerge as one more status distinction among many.

The theory, as it stands, does not provide clear predictions about several important issues: (1) how large will the status distinction be, and (2) will it be of the same magnitude for all social groups, i.e., will it create new intersectionalities by having different effects according to pre-existing status characteristics?

First as to the magnitude of the status distinction, we think, at this point, it must remain an issue for induction rather than deduction. This is not unusual: Sociological theories often imply the direction and existence of a relationship, rather than specifying its size. The logic of Status Construction Theory itself does not imply a specific magnitude, but we can put some bounds on it. For a lower bound, the centrality of the cultural values should matter: Because health and survival are core values and Antibody Positive status is thought to substantially enhance the health and survival of others, the magnitude should be large enough to detect with an ordinary sized sample. But, because it is new, it seems likely to have a smaller impact on a person's overall status than will pre-existing status distinctions associated with work and reward, such as occupational prestige.

Second, as to whether the magnitude is likely to be the same across social groups that differ on pre-existing dimensions of status, there are several conflicting possibilities. (A) A uniform hypothesis suggests that since all members of society value health and survival and since persons in all walks of life who have COVID-19 are equally infectious, being Antibody Positive and hence COVID-free should raise status by the same amount for people in all social groups. (B) By contrast, a scapegoating hypothesis would suggest that “Others” who belong to disadvantaged social groups would experience an overall status loss—this implies that the magnitude of the difference between Antibody Positives and Others in disadvantaged groups will be larger than in advantaged groups, but only because the disadvantaged Others will actually lose status. (C) Alternatively, a niche-differentiation hypothesis would posit that the status gains (or losses) would be conditional on the type of work. Many highly educated people can perform their work effectively from home, so their antibody status is much less consequential to other people's health and safety. Accordingly, Antibody Positives with university or higher education should gain very little status compared to their Other peers. By contrast, Antibody Positive status differences should be large for those whose work requires physical contact (e.g., CNAs, EMTs, childcare workers), near proximity (e.g., receptionists, flight attendants, beauty salon workers), or work in other people's homes (home health aide, house cleaner). All three of these are hypotheses.

In terms of the development of Status Construction Theory, assessing these additional issues will extend the theory in important ways. Examining the magnitude of the Antibody Positive status distinction compared to pre-existing differences will lead to grounded theory about what aspects or characteristics lead to larger or smaller status distinctions. Assessing whether the distinction emerges uniformly across society or whether the effect is amplified or dampened in different settings will provide new insight into the degree to which status construction processes are context-dependent. Moreover, assessing the magnitude and relative importance of some of the additional channels of information about second and third order beliefs that are inescapable “in the wild” will reveal whether expanding the scope of the theory in these directions is useful and will also contextualize the channels that have already been clearly established in experimental settings.

The future? It is plausible to predict that the status distinction will collapse as we draw nearer to an effective vaccine or anti-viral medication. Once there is an established preventative or cure, the unique connection of Antibody Positive status to health and survival vanishes and the public's inclination to overlook the dark side, the substantial proportion of Antibody Positives who acquired their status through deviant behavior, will evaporate. Status construction theory predicts that this status distinction will then shrink considerably, before vanishing entirely when the epidemic is over. Some Antibody Positive people may be able during the epidemic to convert their status gain into other resources that last, but, net of that, Antibody Positive status, in itself will cease to be a status distinction.

Interesting as these extensions are, it is key now to begin by assessing the core model, as laid out in the “Summary” above. At the very foundation is the question of whether hierarchical status beliefs have emerged: How widespread are perceptions that “most people” accord higher status to Antibody Positive people? If so, how does this come about? How large are the impacts of direct experience and first-hand observation—seeing public signs or notices differentiating access by antibody status, being a party to or watching encounters in which antibody status is used to “pull rank”? Besides those, how much does hearsay about such incidents through social networks matter? And does the incidental depiction of such encounters in entertainment and other media influence perceptions of the climate of opinion? How much influence does issue framing and institution building by legitimated authorities have? Answering these questions is essential to assessing how well status construction theory explains public perceptions of majority opinion and the degree to which individuals' behavior is influenced thereby. Through these substantive assessments, analyzing this situation “in the wild” will also cast light on the magnitudes and relative importance of first-order, second-order and third-order inputs forming individuals' status beliefs (their own third order beliefs and expectations). Key next steps in this direction will involve survey research ascertaining perceptions and experiences, observational research evaluating public encounters in different contexts, and content analysis of conventional and social media incidental and focused coverage of such encounters.

## Author Contributions

ME wrote the article. All authors have contributed to the thinking that this article summarizes and have contributed to the organization.

## Conflict of Interest

The authors declare that the research was conducted in the absence of any commercial or financial relationships that could be construed as a potential conflict of interest.
